# Masculinities and self-perceived risk of contracting STIs among young men in Stockholm, Sweden

**DOI:** 10.1093/eurpub/ckac131.574

**Published:** 2022-10-25

**Authors:** A Canabarro, M Salazar

**Affiliations:** Department of Global Public Health, Karolinska Institutet, Stockholm, Sweden

## Abstract

**Background:**

Risk perception is a key factor influencing young men’s health-seeking behavior for sexually transmitted infections (STIs) detection. Men’s risk perception could be influenced by gendered norms embedded in social constructions of masculinities. However, the association between different domains of traditional masculinities and young men’s risk perception - which is key to inform effective health interventions - has not been studied yet. This study aimed to test if young men’s endorsement of different traditional masculinities forms were associated with current self-perceived risk of contracting STIs among young men in Stockholm, Sweden.

**Methods:**

A population-based cross-sectional survey was conducted in 2018 in Stockholm (N = 521 men aged 20-29 years). The Conformity to Masculinity Norms Inventory 46-items tool was used to measure different traditional masculinity domains (Winning, Emotional control, Risk taking, Violence, Playboy, Self-reliance, Primacy of work, Power over women and Heterosexual self-presentation) and overall masculinity. Adjusted multinomial logistic regressions tested the associations between masculinities (overall and each domain) and self-perceived risk of contracting STIs.

**Results:**

Any self-perceived risk of contracting STIs was reported by 39.5% of the sample. After adjusting for confounding factors, endorsing any traditional masculinity behavior was associated with reporting any perceived risk of contracting STIs (RRR 4.9; 95% CI 2.4-10.0). Among the domains, Playboy showed the strongest association (RRR 3.6; 95% CI 2.5-5.1), followed by Risk taking (RRR 1.8; 95% CI 1.3-2.5).

**Conclusions:**

Young men who endorse traditional masculinities behaviors have higher self-perceived risk of contracting STIs, especially those endorsing playboy and risk-taking norms. These findings highlight the need to design policies challenging traditional masculinity behaviors among young Swedish men that can be underpinning their self-perceived STIs risk.

**Key messages:**

• Endorsement of playboy (willingness to have casual sexual partners) and risk taking (willingness to be exposed to risk) masculinity norms were associated with perceiving any risk of contracting STIs.

• Policies challenging traditional masculinity norms among young Swedish men can be designed to influence behaviors underpinning the self-perceived risk for STIs in this group.

